# Advances in Tissue Engineering and Innovative Fabrication Techniques for 3-D-Structures: Translational Applications in Neurodegenerative Diseases

**DOI:** 10.3390/cells9071636

**Published:** 2020-07-07

**Authors:** Federica Rey, Bianca Barzaghini, Alessandra Nardini, Matteo Bordoni, Gian Vincenzo Zuccotti, Cristina Cereda, Manuela Teresa Raimondi, Stephana Carelli

**Affiliations:** 1 Department of Biomedical and Clinical Sciences “L. Sacco”, University of Milan, Via Grassi 74, 20157 Milan, Italy; federica.rey@unimi.it (F.R.); gianvincenzo.zuccotti@unimi.it (G.V.Z.); 2 Pediatric Clinical Research Center Fondazione “Romeo ed Enrica Invernizzi”, University of Milano, Via Grassi 74, 20157 Milano, Italy; 3 Department of Chemistry, Materials and Chemical Engineering “Giulio Natta”, Politecnico di Milano, Piazza Leonardo da Vinci 32, 20133 Milano, Italy; bianca.barzaghini@polimi.it (B.B.); alessandra.nardini@mail.polimi.it (A.N.); 4 Dipartimento di Scienze Farmacologiche e Biomolecolari (DiSFeB), Centro di Eccellenza sulle Malattie Neurodegenerative, Università degli Studi di Milano, Via Balzaretti 9, 20133 Milano, Italy; matteo.bordoni@unimi.it; 5 Genomic and post-Genomic Center, IRCCS Mondino Foundation, Via Mondino 2, 27100 Pavia, Italy; cristina.cereda@mondino.it

**Keywords:** additive manufacturing, scaffold geometry, disease modeling, cell therapy, stem cells, neurodegenerative diseases, 3-D structures, regenerative medicine

## Abstract

In the field of regenerative medicine applied to neurodegenerative diseases, one of the most important challenges is the obtainment of innovative scaffolds aimed at improving the development of new frontiers in stem-cell therapy. In recent years, additive manufacturing techniques have gained more and more relevance proving the great potential of the fabrication of precision 3-D scaffolds. In this review, recent advances in additive manufacturing techniques are presented and discussed, with an overview on stimulus-triggered approaches, such as 3-D Printing and laser-based techniques, and deposition-based approaches. Innovative 3-D bioprinting techniques, which allow the production of cell/molecule-laden scaffolds, are becoming a promising frontier in disease modelling and therapy. In this context, the specific biomaterial, stiffness, precise geometrical patterns, and structural properties are to be considered of great relevance for their subsequent translational applications. Moreover, this work reports numerous recent advances in neural diseases modelling and specifically focuses on pre-clinical and clinical translation for scaffolding technology in multiple neurodegenerative diseases.


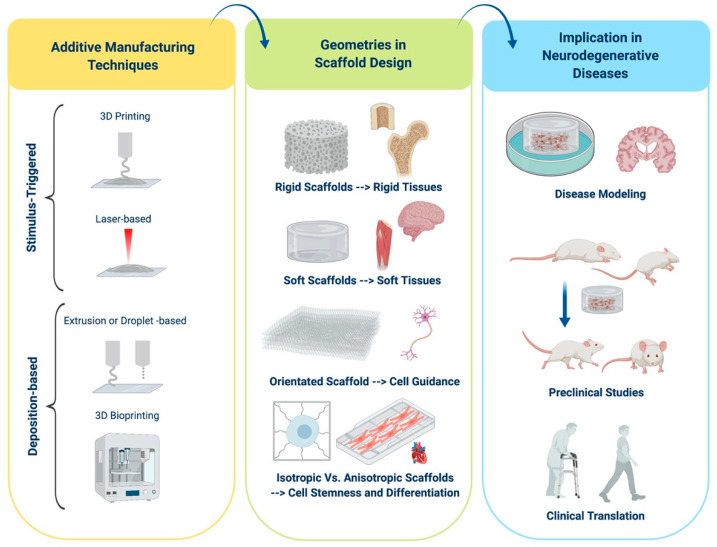


Made in ©BioRender—biorender.com

## 1. Introduction to Scaffold Design

The field of tissue engineering relies on the use of three-dimensional scaffolds as templates for tissue formation [1]. Scaffolds are typically defined as complex 3-D structures whose purpose is to provide a favorable environment for cells’ adhesion and growth, and to give structural support when implanted “in vivo” [2,3]. These structures are gaining more and more relevance in cell biology and tissue engineering as the development of new biomaterials and 3-D scaffolds exhibits a lot of potential in the production of functional 3-D structures with increased biomimetic features [3,4,5].

### 1.1. Scaffold Features

The design of scaffold architecture must be tissue specific in terms of porosity (pore shape and size), morphology (interconnection and fibers’ orientation), and surface topography (shape and roughness) [6]. These features are essential to improve cell homing (adhesion, survival, migration, differentiation) and to facilitate the flow of culture medium (in vitro) or blood (in vivo) through the construct in order to ensure the supply of nutrients and oxygenation [2,6]. When implanted, the engineered scaffold must be biocompatible in order to avoid both immune reactions and inflammatory responses, as well as the toxicity of the products of degradation for biodegradable scaffolds. The scaffold should have equivalent mechanical properties to that of the native tissue, in terms of stiffness and structural stability, as these influence cells’ adhesion, proliferation, and differentiation. Moreover, the scaffold’s degradation kinetics has to be balanced with the new tissue formation [2]. These characteristics are of great importance to adequately support the regeneration process of the recipient tissue or organ [3].

### 1.2. Approaches to Tissue Engineering

**Figure 1:**
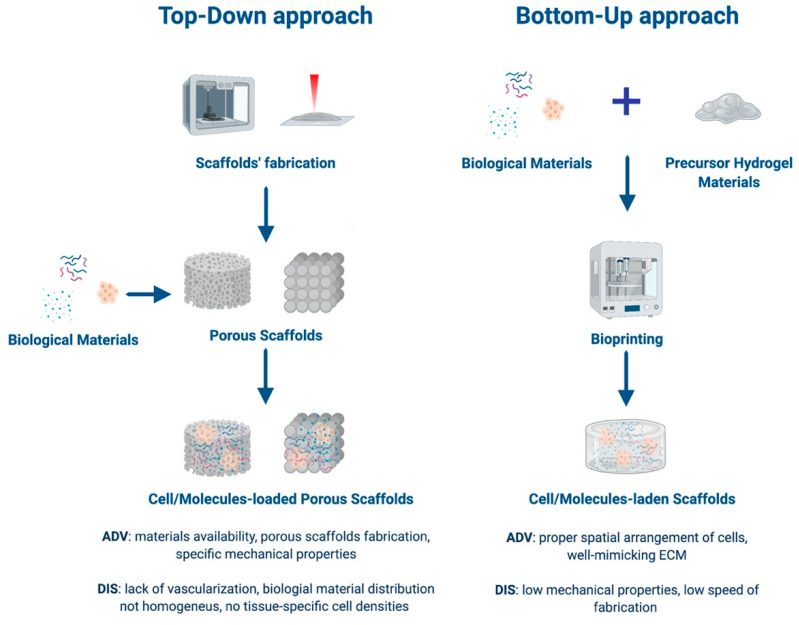
Schematization of the approaches applied in additive manufacturing (AM) techniques. On the left, the top-down approach is shown, which employs AM techniques to produce 3-D scaffolds with the appropriate architecture to guide the formation of the desired tissue. In this case, living cells are seeded on or within the porous 3-D structures. On the right, the bottom-up approach is described, where scaffolding materials, cells, and sometimes also bioactive factors are assembled together, forming building units of several shapes and sizes. Advantages (ADV) and disadvantages (DIS) of each technique are also reported. Made in ©BioRender—biorender.com.

Tissue engineering is mainly based on two approaches: Top-down or bottom-up (Figure 1). The first one employs additive manufacturing (AM) techniques, which are advanced manufacturing processes based on the sequential addition of material, in order to produce 3-D scaffolds with the appropriate architecture to guide the formation of the desired tissue. In this case, living cells are seeded on or within the porous 3-D structures [3,7,8]. The main advantages of top-down strategies are the possibility to use a wide range of processing materials and the production of porous scaffolds with specific mechanical properties according to the applications of interest. On the other hand, the lack of proper vascularization of the construct, the challenges in a homogeneous distribution of multiple cell types, and the subsequent impossibility to achieve tissue specific cell densities represent some serious limitations [3,6,9,10]. In bottom-up approaches, scaffolding materials, cells, and sometimes also bioactive factors are assembled together, forming building units of several shapes and sizes [11]. Using different bottom-up processes, such as hydrogel encapsulation, self-assembled cell aggregation, cell sheets, and 3-D bioprinting, it is possible to achieve constructs with more complex functions [3,12]. Recently, bottom-up approaches have gained more and more relevance because they allow for an optimal control over the spatial arrangement of cells, obtaining an architecture that could strictly mimic the organization of the native tissue [9,12]. However, these processing techniques are often relatively slow, making the assembly of larger tissues challenging. In addition, bottom-up techniques usually use materials with low mechanical properties (e.g., in the range of 0.2–1700 kPa for hydrogels composed of various biomaterials [13]), suitable to reproduce extracellular matrix (ECM) features but limiting the structural aspect of the construct [9]. Both tissue engineering approaches will benefit from the development of innovative AM techniques, which could be helpful in the production of realistic ECM-like scaffolds [3,12].

### 1.3. Classes of Biomaterials

Biomaterials used for scaffold fabrication are usually classified in synthetic polymers, natural polymers, and ceramics [2]. Synthetic polymers are processed using a wide range of assembly approaches as they are not present in nature. Thanks to their great processing flexibility and ease of manipulation, this class of materials is widely used to produce structures with tailored architecture, appropriate mechanical properties, and controlled degradation features according to application-specific requirements [14,15]. These features can be achieved by acting on the molecular weight distribution of the material and varying the monomer composition of copolymers [16]. A reduced bioactivity, meaning a lack of interaction between the tissue and the synthetic scaffold, derived from the inability to combine the material with bioactive molecules because of low affinity, is the main limitation of the synthetic polymers [2,3]. Despite a minimal immune response, polylactic acid (PLA), polyglycolic acid (PGA), and poly(lactic-co-glycolic acid (PLGA) are among the few synthetic polymers Food and Drug Administration (FDA)-approved for clinical applications [17,18,19]. In particular, PLGA, thanks to its adaptable design and the capacity to provide bioactive signals to enhance cell–biomaterial interaction, is one of the most common synthetic polymers in clinical use for bone and cartilage tissue regeneration [20]. Natural polymers are convenient in tissue engineering for their excellent biocompatibility, and their ability to promote cell adhesion and growth [2]. Biological materials, such as alginate, hyaluronic acid, and chitosan, are polysaccharide based or a derivative from proteins, such as collagen, fibrin, and silk. This kind of polymers are widely available in nature and their biodegradability, in addition to their bioactivity, closely mimics that of the ECM [21]. 

Natural polymers promote minimal inflammatory and immune responses [3]. However, whilst being processed, the materials often lose their biological functions, making the fabrication of scaffolds with homogeneous and repeatable structures challenging [2]. In addition, natural polymer-derived scaffolds generally have poor mechanical properties, which limit their application in mechanical load-bearing anatomical sites, such as in hard tissue regeneration. The combination of natural biomaterials with synthetic ones to produce hybrid scaffolds of multiple phases is an attractive solution to overcome all these limitations [3,22,23]. Ceramic materials can be classified as nearly inert (alumina and zirconia), bioactive (based on bioactive glass), and resorbable (based on alpha and beta tricalcium phosphate) [3]. Bioceramic scaffolds are commonly used in bone tissue regeneration thanks to their biocompatibility and bioactivity, along with the possibility to achieve hierarchical structures at micro and nano scales whilst mimicking the native tissue well [24]. They are characterized by high mechanical stiffness and low elasticity. Their brittleness and tricky machining processability into specific shapes, in addition to the mechanical properties previously underlined, limit their clinical applications [25]. Moreover, the degradation kinetics of ceramic scaffolds does not match the new bone formation [2,3]. The assembly of polymers and ceramic materials promotes greater flexibility to the final construct, rendering it more similar to the native tissue, offering biological benefits as well as an improvement of the mechanical properties [26]. To support and improve the regeneration of hard tissues and to produce parts with highly complex geometries, metallic materials are commonly used. They can be of relevance in the field of regenerative medicine and are mainly employed in the production of medical implants [7]. Another innovative strategy relies on the use of decellularized ECM as a template for tissue regeneration. The decellularization process relies on the enzymatic, chemical, or physical removal of the entire cellular part, whilst maintaining the original ECM’s structural integrity and tissue’s vasculature network, thus preserving the design features at the micro and nano scale [27]. For these reasons, ECM-based scaffolds provide an ideal environment for cell adhesion and growth, similar to that of the native tissue [28]. On the other hand, the low availability of donors and the accelerated degradation, which leads to the lack of a cell repopulation and tissue remodeling, are limitations that need to be overcome before ECM-based scaffolds become applicable [3,29].

## 2. Fabrication Techniques

**Figure 2:**
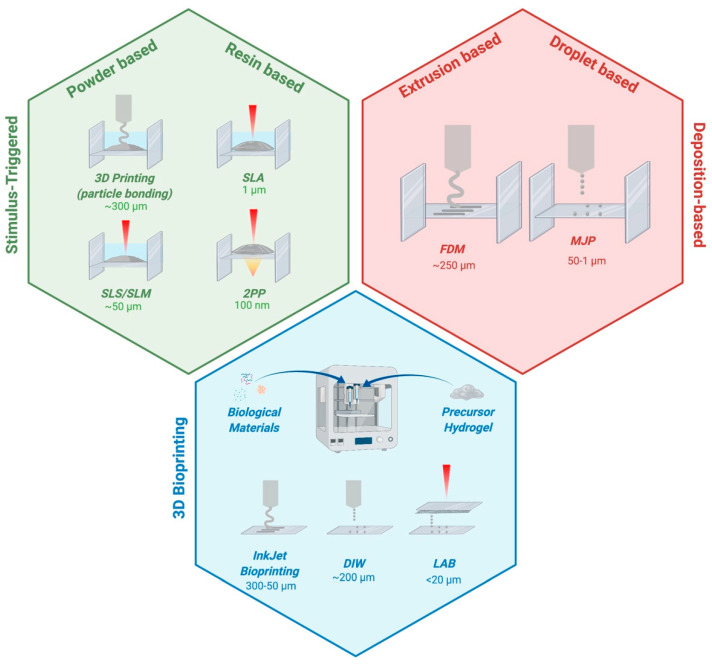
Summary of the three main categories of additive manufacturing techniques for scaffold fabrication. The top left hexagon reports the different stimulus-triggered approaches, subdividing them into powder based (3-D printing and SLS/SLM) and resin based (SLA and 2PP). The top right exagon reports the two main categories of deposition-based techniques: extrusion based (FDM) and droplet based (MJP). The lower central hexagon refers to the different types of 3-D bioprinting: InkJet Bioprinting, DIW, and LAB. Made in ©BioRender—biorender.com.

Additive manufacturing (AM) techniques are proving to have great potential in the fabrication of precision biomaterials as they overcome the limits of traditional subtractive manufacturing techniques, typically based on material removal from a solid block to generate the final construct [6,7,30,31]. Indeed, AM techniques are free-form processes as they rely on sequential addition of material to form the 3-D structure. These advanced processing methods allow freedom of design, the production of scaffolds with complex geometries, and they could provide patient-specific fabrications. These are at the basis of personalized medicine, where the curing agent is developed to fit the immune compatibility and the therapeutic need of the patient [7,31,32]. AM techniques could be classified as stimulus-triggered AM and deposition-based AM, according to the introduction of a trigger or the direct deposition of the material during the process. In stimulus-triggered AM, the biofunctionality of the final construct could be influenced by the nature of the stimulus applied; for this reason, cells and bioactive molecules are usually added post fabrication, making this a typically top-down approach. Despite the high processing speeds and high spatial resolutions achieved, stimulus-triggered AM does not allow the production of multi-material scaffolds. On the other hand, deposition-based AM enable the direct fabrication of cell-laden constructs, and cell survival is ensured by properly controlling process parameters and material design constraints [7]. These techniques were born as top-down approaches. Even if these approaches are still utilized, new technologies, grouped as “3-D bioprinting techniques”, were also developed and represent an exception, as they allow for the direct encapsulation of biological materials and are thus bottom-up approaches. Deposition-based AM also presents with some disadvantages, such as a loss in resolution and a relatively slow speed of processing [7,33,34]. A summary of these techniques is illustrated in Figure 2 and a more comprehensive overview is reported in Table 1.

### 2.1. Stimulus-Triggered Approaches

Stimulus-triggered approaches rely on the introduction, during the fabrication process, of a trigger to induce the material’s (liquid or powder) solidification and organization in a specific spatial location, defined voxel, in order to form the desired 3-D structures [7]. These techniques can be classified in 3-D printing based on particle bonding and laser-based AM based on the used trigger [7].

#### 2.1.1. 3-D Printing Based on Particle Bonding

The 3-D printing technique based on particle bonding relies on the ejection of a binder solution on the top of a powder bed, in accordance with a software 3-D model [35]. When the binder is printed, a thin 2-D layer of material is solidified and the powder support moves down as a fresh layer of powder is deposited. This process is sequentially repeated binding a layer to the next leading to a “layer by layer” formation of complex 3-D structures that will later be subjected to a post fabrication treatment in order to remove the embedded unprocessed powder [6,7,8,36]. This kind of 3-D printing allows the production of porous scaffolds, characterized by channels and overhanging features, with control over the pore architecture by operating on the region of bounding. There is also control over the micro-porosity, which depends on the space between the granules of powder [36]. Moreover, it is possible to use one-component powder or different powders blended together. On the other hand, the spatial resolution achieved is ~300 µm and the pore size is limited by the size of the powder used. As with other powder-based techniques, 3-D printing based on particle bonding provides a rough surface to the fabricated scaffold, a feature that may be relevant for cells–material interaction [6].

#### 2.1.2. Laser-Based Techniques

Laser-based AM are direct laser writing (DLW) techniques, which employ focused light as a stimulus to solidify the material in the specific voxel of the 3-D space [7]. These techniques are based on the excitation of electrons in the atoms and molecules of the fabrication material, induced by photon absorption, or on the photopolymerization phenomenon, which occurs when photons are absorbed by a photo-initiator molecule in the material-forming free radicals [37]. In the first case, energy is converted into heat and, at low energy, localized heating results in sintering or melting of the material, maintaining intact bounds between molecules and avoiding material detachment [38,39]. In the second case, radicalized molecules promote a series of polymerization reactions in the target material [40]. The most important parameter in DLW techniques is the laser wavelength, which, acting on the laser–material interaction, defines the absorption and the scattering of laser radiation in the material. Solid-state lasers, meaning that the active laser medium is solid, usually work from ultraviolet (UV) to infrared (IR) wavelengths and can operate in a continuous wave or in pulsed mode [41]. These two modes of operation differ for the output energy, which is, in the continuous case, constant over time, compared to the pulsed one in which higher energies are involved, emitted over short time pulses [41]. Using continuous lasers, parameters to keep in consideration are the power as the total energy per unit time; the irradiance as the power per unit area; the numerical aperture of the objective used, which influences the final resolution of fabrication; and the duration of laser exposure defined as the time during which the laser emits radiation [37]. In the case of pulsed lasers, important parameters are also the pulse duration, the pulse energy, the repetition rate, the average and peak power, and the fluence, defined as the amount of energy divided by the sample surface per unit area [37]. The use of a laser beam provides focused spatial energy, allowing materials at the micro and sub-micro scale to be processed, creating micro and nano patterns with self-supporting features. For this reason, the main advantage of DLW techniques is the high spatial resolution achieved in addition to the possibility of building complex 3-D architectures, which are of great importance in the field of personalized medicine [41,42]. Moreover, it is possible to use different materials depending on the application and, in some cases, incorporate biological components within the material, avoiding its deterioration [37]. The most investigated laser-based AM techniques are selective laser sintering/selective laser melting (SLS/SLM), stereolithography (SLA), and two photon polymerization (2PP).

##### Selective Laser Sintering and Selective Laser Melting

Selective laser sintering (SLS)/selective laser melting (SLM) are both powder-based DLW techniques but differ in the process of 2-D pattern formation, which leads to powder sintering or melting [39]. These approaches rely on the use of continuous lasers, such as CO_2_ lasers involving high power and long pulse lasers, as they are thermally activated techniques [43,44]. The laser beam is focused on a thin layer of powder, which is locally heated by the electromagnetic radiation emitted by the laser, at a temperature at which the granules of the powder sinter or melt together, forming solid 2-D patterns. Moving the adjustable table on which the powder lies and adding fresh powder over the solid 2-D layer previously formed allows the fabrication of 3-D structures; this process is repeated until the structures are complete, leading to a layer-by-layer fabrication. At the end of the process, it is necessary to remove the un-sintered or un-melted powder from the 3-D construct, manually or with brushing and powder blasting, thus avoiding the use of organic solvents [37,41]. The main advantage of SLS/SLM processes is the possibility to construct overhanging regions with no need for support structures thanks to the unfused powder outside the sintered/melded one that, remaining within the fabricated volume, acts and supports material. Using a focused laser SLS/SLM AM achieves a spatial resolution of ~50 µm and the laser’s high energy allows the processing, not only of polymers, but also ceramics and metals, which need to be in powdered form [45]. However, these techniques are characterized by temperatures higher than 37 °C, which limit the direct incorporation of biological materials during the SLS/SLM processing. In addition, the mechanical properties of the final construct, its surface accuracy, and the control of the porous interconnected architecture may be affected by the material properties and by the parameters of the process [34]. Based on the same fabrication process of SLM, electron beam melting (EBM) differs from the previous technique for the use of an electron beam as a power source instead of a laser beam. In particular, EBM is preferable for the production of metallic constructs [34].

##### Stereolithography

Stereolithography (SLA) is a resin-based DLW technique based on the phenomenon of single photon absorption [34]. This technique involves the use of a continuous wave laser at the ultraviolet wavelength with relatively low energy to promote the polymerization of a photosensitive resin [37,42]. The UV light interacts with photoinitiator molecules inside the resin and the presence of the chain precursor in the same resin allows the release of free radicals, initiating the polymerization. Resins containing acrylate, epoxy, urethane acrylate, or vinyl ether functional groups are typically used [46]. Following the computer-aided designed geometry, the UV laser moves in 2-D over a reservoir containing the resin. The polymerization occurs a few µm below the surface, in the regions of the laser–resin interaction. The fabrication of the final 3-D construct happens layer by layer, relying on the down motion of the polymerized structure within the resin vat, after the selective polymerization of a given layer, which will be recoated with unpolymerized resin [7,32]. The displacement is equal to the thickness of the last polymerized layer, and the depth of penetration of the UV light allows the adhesion between layers. After fabrication, the final construct has to be placed in a developing solution to remove the unpolymerized resin, but some monomers of liquid resin can still be trapped within the structure, inducing toxicity. To overcome this issue, a post curing step is necessary, which consists in exposing the 3-D construct to high-intensity UV light for up to 2 h in order to polymerize the whole material, reducing its toxicity and increasing its hardness [37,42]. In the past years, SLA was the most used laser-based AM technique thanks to the low cost of the equipment and the relatively high processing speed due to the use of a continuous laser at low energies [34,37]. The spatial resolution achieved by SLA processes was limited by the diameter of the laser beam (~250 µm), but recent improvements in most commercial systems enable the production of scaffolds with a resolution greater than 50 µm (up to 1 µm), and with well-interconnected and regular pores [37,41]. Moreover, SLA techniques avoid the use of high processing temperatures, allowing the incorporation of biological material within the structures during the fabrication process. In this case, the effect of laser energy, the toxicity of the photoinitiators, and the DNA damage induced by UV light have to be evaluated and could be a concern. To overcome these problems, visible light-based SLA are currently being investigated. SLA techniques show some limitations regarding the material selection due to the high thermal coefficient of expansion of SLA-compatible resin and the possibility of distortion and shrinkage of SLA-processed materials [33,37].

##### Two Photon Polymerization

Two photon polymerization (2PP) is a resin-based technique of DLW that, in a similar way to SLA, promotes the curing of a photosensitive resin, inducing chemical reactions between chain precursor and photoinitiator molecules, thanks to the excitation of the latter [47]. Differently from SLA, which is based on a single photon absorption, 2PP techniques rely on the near simultaneous absorption of two photons to excite photoinitiator molecules; this electronic excitation corresponds, in terms of energy, to the excitation achieved by a single photon, which possesses a much higher energy [48]. 2PP uses focused near-infrared (NIR) femtosecond laser pulses (with a wavelength of ~800 µm) to induce the photopolymerization that occurs in regions where the energy exceeds the photoinitiator excitation threshold. The two photon absorption phenomenon shows a non-linear laser–material interaction due to a material response proportional to the square of the photon intensity; in this way, the reaction is greatly enhanced at the focal point, allowing feature sizes below the diffraction limit of the applied light. Thanks to that, 2PP can achieve a sub-100-nm spatial resolution, much higher than that of the other DLW techniques [42,49,50]. In addition to the use of a femtosecond laser, a 2PP system relies on the use of a high numerical aperture microscope objective in order to focus the laser beam and scale the feature sizes on Galvano mirrors that guide a translational platform to scan the beam in the X and Y directions, and on a piezoelectric system in order to shift the plane of resin or the objective holder in the Z direction. 2PP-compatible resin has to be UV curable and the advantage is that many materials, commonly used in 2PP, are transparent to NIR-wavelength light, making materials processed in 2PP widely available and inexpensive [37]. Moreover, the process can be set up in a conventional environment that does not require specialized equipment or cleanroom facilities [51]. The main advantage of 2PP technology remains the higher resolution achieved, down to the subcellular-length scale, with the possibility to fabricate a 3-D structure with a large range of features sizes, allowing, at the same time, minimization in processing time and costs. However, the restrictions due to the objective’s working distance could limit the 2PP processes in terms of scalability compared to single photon absorption techniques (e.g., SLA) [42]. 

Two more DLW techniques exist in addition to the laser-based approaches previously described, but they fall outside the aim of this review as they are destructive techniques, and not AM approaches. Both involve the use of the laser ablation phenomenon and they are the laser machining technique based on the removal of a small amount of material from the bulk, and the matrix-assisted pulsed-laser evaporation (MAPLE) technique in which the material is transferred from a coated ribbon to a substrate [37].

### 2.2. Deposition-Based Approaches

Deposition-based approaches rely on the local and direct deposition of the material [7]. The solidification of the final 3-D construct occurs during or immediately after the material’s deposition. These approaches can be classified in extrusion-based (e.g., fuse deposition modelling) and droplet-based techniques (multijet printing) on the base of the principle of operation [7,52].

#### 2.2.1. Extrusion-Based Techniques: Fuse Deposition Modelling

Extrusion-based techniques allow the building of a 3-D construct, which relies on the extrusion of the processing material in a continuous flow. The advantage in using this kind of approach is that the processes involved are mechanically simple and inexpensive [34]. The most common and accessible extrusion-based technique is fuse deposition modelling (FDM) due to the low costs of production and the easy principle of fabrication [53]. FDM could process any material, which can be in a filament form, and is based on the heating of thermoplastic polymers over their temperature of melting; the extrusion head moves in the Z direction, extruding the processing material as filament in a layer-by-layer fabrication thanks to its computer-controlled locations of deposition and its solidification upon cooling [7]. Despite the wide range of FDM-compatible thermoplastic polymers, the need to apply high temperatures to melt the polymers’ filaments is an impediment to the direct encapsulation of biological materials during the process [7,32]. Even so, the change in the material’s properties leads to a need to recalibrate all the setting parameters. The spatial resolution achieved is ~250 µm, which limits the dimensional accuracy of the FDM-fabricated parts. However, this reduced resolution is compensated by the relatively high processing speed and by the lack of a need for toxic solvents as the binding between each layer occurs through thermal heating [32,41]. To overcome the restrictions in the input material properties, precision extruding deposition (PED) techniques could be taken into consideration. Differently from the FDM process, the PED system does not require filament preparation and the processing material is provided to the system in a powder form, subsequently liquified, and finally extruded though a nozzle. Moreover, in order to have the possibility to fabricate high-density metallic and ceramic parts, useful, for example, for the manufacturing of porous scaffolds for bone tissue regeneration, multiphase jet solidification (MJS) is an alternative to the FDM technique [34].

#### 2.2.2. Droplet-Based Techniques: Multijet Printing

Droplet-based techniques allow the production of the final structure relying on the deposition of liquid material in droplet form instead of continuous flow as in extrusion-based ones [35,54]. Material solidification occurs after its deposition and could take place via cooling (crystallization), chemical changes (cross-linking), or solvent evaporation [35]. The most common droplet-based approach is multijet printing (MJP), which is based on the use of several heads placed on a jetting head, which, moving in the X and Y direction, deposits tiny droplets of UV-curable resin, promoting material layer formation on the build tray. Along the jetting head there are some UV bulbs that, after each layer is built, harden the deposited material and, shifting down the tray in the Z direction, allow the deposition of the next layer [55]. Control of the rheology, meaning the control of the viscoelastic flow behavior of the printing materials, is a crucial aspect of jetting techniques; the behavior of droplets and the liquid jet is affected by the physical properties of the chosen material, resulting in a restriction on jet-compatible materials, raising their cost. For these reasons, MJP is more suitable for large-scale production [34,35]. On the other hand, MJP has the great advantage of achieving a spatial resolution comparable to that of laser-based systems [34].

### 2.3. 3-D Bioprinting Techniques

Recently, some deposition-based AM technologies have been developed for the fabrication of cell-laden biomaterials grounded on the direct encapsulation of biological materials, such as living cells and active molecules into formed 3-D constructs [7,56]. This kind of application has encouraged the evolution of the so-called bioprinting techniques.

Three-dimensional bioprinting techniques are based on the direct encapsulation of biological material during the fabrication process, giving rise to cell-laden biomaterials [33]. The most suitable materials used in bioprinting approaches are hydrogels, thanks to their ability to mimic the ECM and to provide a proper environment for cells, facilitating their migration, proliferation, and differentiation [7,56,57]. The use of hydrogels to carry cells and/or bioactive molecules defines them as “bio-inks” (hydrogels combined with biological materials) and involves the need to fulfil specific requirements regarding their rheology, in terms of viscoelastic properties, such as viscosity, and post-curing behavior, according to the proper bioprinting technique, to fabricate functional 3-D constructs [56,58]. When the final construct has been printed, processed, and the cells are alive within, it must achieve appropriate mechanical, physical, and biological properties. The main limitation of hydrogel-based AM technologies is the low mechanical properties, leading to difficulties in the fabrication of larger stiff structures [7,59,60]. The three most common 3-D bioprinting techniques are inkjet bioprinting, direct ink writing, and laser-assisted bioprinting, which differ for the deposition technique.

#### 2.3.1. Inkjet Bioprinting

Inkjet bioprinting is a non-contact technique in which droplets of bio-ink are dispensed through a small orifice and precisely positioned on a substrate or a collective platform according to digital instructions [33,53]. Inkjet bioprinters differ for the physical mechanism of dispensing, which can be thermal, piezoelectric, or pneumatic microvalve based. The thermal mechanism relies on heating of the printhead generating the pulse that promotes the ejection of small vaporized bubbles. The localized heating in a thermal printer lasts for a very short time, but it can still cause a stressful condition for the deposited cells [56,61]. In the piezoelectric printer, a piezoelectric actuator generates an acoustic wave, which mechanically breaks the bio-ink into small droplets, forcing their ejection from the nozzle due to a transient pressure [56]. The pneumatic microvalve-based printer regulates the bio-ink ejection through a constant pneumatic pressure [21]. Inkjet bioprinting techniques are able to achieve a spatial resolution between 300 and 50 µm, and moreover, the quality of the printing may be affected by cellular aggregation within the hydrogel, which can induce changes in droplet formation and trajectory [62]. Moreover, one of the main restrictions of these techniques is the low upper limit of viscosity for the ink involved, hindering the processing of high viscous material and the building up of 3-D constructs and overhanging structures [56,63,64]. For this reason, inkjet bioprinting is mainly used for small scaffold production, with the capability to print a single cell per droplet [21,33]. The use of printheads with multiple nozzles has been investigated in order to increase the processing speed and allow the production of larger-scale constructs [65]. However, the small droplet size and the limitations in the viscoelastic properties of the materials involved, such as the low mechanical properties of the bio-ink, make the application of this technology challenging for larger-sized productions. On the other hand, inkjet bioprinting’s flexibility to print multiple bio-inks makes possible the production of complex multiphase tissues [21,33].

#### 2.3.2. Direct Ink Writing

Direct ink writing (DIW) is an extrusion approach, which implicates filament printing instead of droplets. It is based on the bio-ink extrusion through a printhead driven by piston, screw, or pneumatic pressure mechanisms in order to build up 3-D structures [8,33,66]. Piston-driven and screw-driven extrusion mechanisms are mechanical-based systems, which can induce cell apoptotic effects due to the pressure drops generated at the nozzle [67]. Pneumatic-based extrusion mechanisms are more suitable for work with highly viscous inks, as they are the only ones able to maintain a filamentous structure after deposition. Even so, mechanical-based systems promote more direct control over the ink flow and more spatial control on the ink ejection [67]. The high speed of fabrication and the ability to print at very high cellular densities within the inks are the main advantages of DIW techniques. DIW also allows control over the deposition and distribution of cells within the inks, and an excellent structural integrity due to a continuous deposition of the bio-ink [8,68]. For all these reasons, the application of DIW technology for scaffold fabrication is of great relevance, despite a spatial resolution of ~200 µm, which is lower compered to inkjet and laser-assisted bioprinting [21,33]. One of the most common extrusion techniques able to produce polymeric non-woven fibers is electrospinning, which, allowing the possibility to incorporate biological compounds within processing materials, is able to fabricate cell-laden structures, with hydrogel based becoming a DIW technique [7]. Direct writing electrospinning (DWE) relies on the application of a high electric field to create an electrically charged jet of polymer, which is ejected from the nozzle of the Taylor cone and travels toward the collecting plate [67]. DWE enables the fabrication of 3-D structures, single-fiber or multi-fiber, with a well-controlled geometry [69]. Despite this, there is a need to carefully consider the rheology of the extruded material and the desired features of the final scaffold, as the main limitation of this technique is the difficulty to set the process parameters [7,67].

#### 2.3.3. Laser-Assisted Bioprinting

Laser-assisted bioprinting (LAB) is a droplet-based and scaffold-free technique based on the use of a laser as the energy source to deposit biomaterials on a substrate [33,70]. LAB consists in a pulsating laser, a donor slide coated with the target biomaterial to support it, and a natural or synthetic receiver slide to collect and support the printed material. The donor slide is also coated with a thin gold/titanium layer, which, via laser induction, promotes a vaporization effect on the biological materials, propelling it onto the receiver slide in droplet form. To maintain cellular viability, the receiver slide is covered by a biopolymer or cell culture medium [71,72]. The precursor biomaterial used is hydrogel and the nozzle-free approach enables the use, not only of mid-range-viscosity bio-ink, but also of high-viscosity ones [66]. Moreover, the main advantage of LAB approaches is the possibility to achieve high resolutions greater than 20 μm, maintaining a high activity for encapsulated cells, and the ability to control the features of the ink droplets and their delivery properties [33,71]. Even so, the resolution and the final mechanical integrity of the construct may be affected by the hydrogels’ viscoelastic properties and the layer thickness of the precursor biomaterial, by the energy of the laser pulse, and by the organization of the desirable structure [33,71,73]. 

Future developments in 3-D bioprinting techniques require the combination of different approaches in order to provide the accuracy in cell placement and resolution of inkjet bioprinting and LAB, and the processing speed and greater mechanical integrity of DIW.

## 3. Geometries

### 3.1. Specific Geometry in Scaffold Design

Depending on the different manufacturing techniques, scaffolds can present different degrees of stiffness and different geometries [74]. In particular, scaffolds must be designed to reproduce the stiffness of the native tissue to regenerate and transplant, and they must present a resolution suitable at the cellular scale [75]. The nano or micro-topography and the specific geometry of different biomaterials induce, at the single cell level, different cytoskeletal tensional states, given by the intracellular actomyosin contractility and by the reaction forces exerted by the surrounding substrate [76]. Indeed, different external stimuli and different geometries can influence specific cellular responses. It is thus necessary to fabricate a scaffold, which mimics the tissue’s physiological environment. The traction forces exerted by the cell depend on the specific scaffolding substrate and produce a different response inside the cell nucleus, resulting in an altered gene expression [77]. Indeed, the study of mechanotransduction allows the understanding of how cells respond to external stimuli [78,79]. 

Several studies have been conducted to investigate the influence of scaffold design parameters on the cell’s mechanical and biological responses [74,80,81,82,83,84,85]. Depending on the specific tissue regeneration, the biomaterial features and geometries of the scaffold were optimized, suggesting sophisticated micro-architectures for unit cell scaffolds [86]. Not only the Young’s modulus of the biomaterials, which represents the index of their stiffness, must be considered to design the scaffold but also the porosity, and the pore size and shape are fundamental parameters to optimize the scaffold performance [87,88].

### 3.2. Techniques Employed for the Fabrication of Rigid Scaffolds

SLM, SLA, 3-D bioprinting, and fuse deposition modelling, described in the previous paragraphs, are the most common techniques used for the fabrication of rigid scaffolds [89,90]. In particular, scaffolds with a high Young’s modulus are used for bone regeneration, in order to simulate the rigidity of the bone in vivo [91]. Indeed, these techniques have a specific and controlled geometry that allows for a higher resolution. In the field of bone regeneration, many researchers focused their attention on the geometries of the scaffolds’ pores. Specifically, important matrix parameters are the pores’ size and shape, and their interconnectivity [92,93]. With the support of algorithms and computer-based models, it was possible to identify the optimal range of pore dimension between 500 and 1000 μm and specific tetrahedral and octahedral geometries fabricated with the SLM technique [94]. In addition, Stoppato et al. demonstrated that, even if a specific geometry is required, a random distribution of the pores seemed to be more suitable for a bone tissue regeneration application, with osteoblasts producing a collagen architecture similar to the natural matrix [95]. Moreover, the pores’ distribution and interconnectivity strongly influence the cells’ ability to proliferate and differentiate [96].

### 3.3. Techniques Employed for the Fabrication of Soft Scaffolds

As previously described, most of the scaffolds with a higher stiffness are compatible with bone regeneration but of great interest are also the scaffolds for the regeneration of soft tissues. The two most common applications of soft scaffolds are muscle and nerve regeneration, where the architecture and the spatial organization of the entire scaffold are necessary. Different fabrication techniques, such as bioprinting and in particular inkjet printing, are suitable for soft materials and specifically for the regeneration of soft tissue [97,98]. In particular, in muscle tissue where there is a strong structure–function relationship, the ability to control the geometry for tissue implantation is essential [99]. The necessary parameters to consider are represented by surface roughness, pores, grooves, walls, and pillars, which can be used to guide cell behavior in terms of adhesion, alignment, and motility [100]. Indeed, the contact guidance for cell directionality and the advanced manufacturing microfabrication permit polymeric micro-structured substrates to be obtained that influence myoblast and myotube formations [100]. Furthermore, tridimensional scaffolds with a micropatterned array can mimic the aligned architecture of the natural skeletal muscle tissue, enhancing tissue regeneration [101]. 

In addition to polymer scaffolds, naturally derived hydrogels have several properties that guarantee the functionality of the muscle tissues. For smooth muscle cell differentiation, it is also possible to consider micropatterned biomaterial-based hydrogel platforms, where mesenchymal stem cells can be induced to differentiate, following precise patterns [102]. Moreover, other studies involved different approaches that utilize photolithographic patterning of hydrogels, enabling a relatively fast layer-by-layer assembly of cells with a controllable geometry and size [103,104].

The same fabrication techniques used for soft scaffolds and hydrogels can be relevant for the fabrication of structures aimed at enhancing the features of the neural tissue. In this case, the structure of neural scaffolds is extremely important for the efficacy and has significantly advanced in recent years [105]. To this end, innovative scaffolds, such as multichannel scaffolds and grooved substrates, have been developed in order to enhance the directionality of growing neuronal processes [106,107]. In this case, the dimension of the guidelines is precise and governs the elongation of the cells with an anisotropic tension state [108].

### 3.4. Structure to Function: Importance of Geometry in Enhancing Cellular Features

The concept that rigid scaffolds are necessary for rigid tissue regeneration and soft scaffolds for soft tissues can be partially overcame when trying to enhance specific cellular features. This is extremely relevant for stem cells’ proliferation and stemness maintenance where the precise and controlled geometry of the scaffold is necessary [109]. The difference in this case is the clinical translation, more focused on the amelioration of stem cells features as therapeutic agents rather than whole tissue re-implantation. To this end, an innovative study inspired by the natural stem cell niche has led to the development of a new stem cell culture system named “Nichoid”. This engineered scaffold is fabricated by 2PP and it is composed of a three-dimensional succession of grids and columns able to create perfectly defined pores at the micrometric scale, ensuring the optical accessibility. Cells expanded inside the Nichoid are subjected to isotropic mechanical stimuli driven by the cytoskeleton [110,111,112,113]. Typically, if the traction forces have a similar magnitude at varying orientations (i.e., isotropic cytoskeletal tension), the cellular nucleus tends to maintain a roundish morphology [114]. For this reason, when cells are grown inside the Nichoid they tend to maintain a round nucleus, similarly to the stem cells’ physiological morphology [77]. 

Conversely, in some cases, anisotropic stimuli are required for a specific commitment differentiation. Indeed, the benefit of scaffold anisotropy was evident with human-induced pluripotent stem cell-derived cardiomyocytes, where parallel-aligned polymer scaffolds can provide contact guidance to cells to reorganize cellular orientation and differentiation [115]. Moreover, Zhang and colleagues applied biomechanical and biochemical stimuli to mesenchymal stem cells seeded into a biomimetic scaffold to induce the differentiation of fibrochondrocytes, resulting in physiological anisotropy in the engineered meniscus [116].

### 3.5. Development of Optimal Scaffolds for the Neural Tissue: A Role for Geometry and Stiffness

The brain is one of the most complex organs to cure and mimic, and in this sense advances in scaffolds development could be of great relevance in order to enhance specific neural features. Indeed, nano-structured scaffolds are a promising strategy to promote axon regeneration, needed for the therapy of neurodegenerative diseases [117]. Moreover, the scaffold’s fibers ensure the directionality of neurite outgrowth and the alignment of neural cells, as observed by cellular elongation and neurite differentiation when these are used [118]. Indeed, Friecke, in 2011, developed different nanostructured patterns by microcontact printing using laminin/poly-l-lysine (PLL). These 3-D structures were investigated for their impact on neurite growth and axon guidance in embryonic rat cortical neurons [119]. Using the same technique and the same materials, another interesting study developed by Jang et al. analyzed 10 different types of micro polygons, ultimately observing that the geometry of the scaffold strongly influences the development of a cultured neuron [120]. In 2017, Kim et al. investigated a different technique, termed electrospinning, in order to develop a 3-D connected artificial neuronal network within a nanofiber-microbead-based porous scaffold. This inspiring scaffold allowed substantial neurite outgrowth in a vertical direction [121]. 

Another feature that needs to be enhanced for neural tissue mimicking and therapy is neural differentiation. Not only the geometry of the scaffold but also its stiffness modulation could influence this process. Indeed, physiologically, neuronal growth and neural network activity are strongly influenced by the mechanical properties of the surrounding ECM [122]. Controlling the scaffold’s features in order to resemble the ECM is crucial for enhanced neural differentiation. One research work reports that, specifically, neuronal differentiation was favored in the softest surfaces with a Young Modulus of 1 kPa, whilst oligodendrocyte differentiation was enhanced in stiffer scaffolds (>7 kPa), and lastly astrocyte differentiation was only observed on <1 and 3.5 kPa surfaces [123]. Indeed, Her et al. showed that mesenchymal stem cells can differentiate into the neuronal lineage in a substrate that present a Young modulus of 1 kPa, while they transformed into glial cells when this parameter is 10 kPa [124]. Moreover, a recent work has shown that the physicochemical properties of the alginate/collagen blend could resemble the ECM microenvironment, influencing neuronal-specific gene expression [125]. In particular, it was shown that oligodendrocyte differentiation and maturation in vitro is enhanced by substrates within the reported range of stiffness of the brain [126]. Finally, Saha et al. developed a synthetic interpenetrating polymer network, which creates a highly mechanically and chemically stimulating environment for multipotent neural stem cells to control their proliferation and differentiation [127]. 

## 4. Scaffolds for Neural Diseases’ Modeling

The brain is difficult to access, susceptible to damage, and complex, making it one of the hardest organs to be studied. This poor understanding of the brain leads to a lack of effective treatments for several neurodegenerative diseases, such as Alzheimer’s disease (AD), Parkinson’s disease (PD), and amyotrophic lateral sclerosis (ALS), but also for acute or traumatic Central Nervous System injuries, such as acute ischemic stroke (AIS) and spinal cord injury (SCI). New methods for a realistic culture of neural cells are needed, and in particular in vitro 3-D cultures represent a promising tool to reconstruct the complex structure and function of the human brain [128]. Traditional monolayer cell cultures cannot mimic tissue architecture, mechanical and biochemical cues, and cell–cell communication. On the contrary, 3-D cell culture systems aim to mimic the living tissue, providing a more physiologically relevant environment [129]. Many natural or synthetic materials can be used to engineer the neural tissue in a 3-D in vitro model, but also functionalization with specific peptides can improve the adhesion, proliferation, and differentiation of neural cells [130,131]. Specifically, two innovative approaches that can be used for neural diseases’ modeling are decellularized scaffolds and hydrogel-based biomaterials. 

### 4.1. Decellularized Scaffolds

An interesting approach to create highly biocompatible bio-inks is decellularization, which consists in removing the cellular content from animal and human-derived tissues. This method allows the production of tissue-specific ECM scaffolds that can more accurately recapitulate the native matrix [132]. These bio-inks have an enormous potential for in vitro modeling of neurodegenerative diseases phenotypes and for evaluating the tissues’ responses to new potential drugs [132]. A potential issue is represented by the complexity of the brain tissue, such as the diverse conditions in terms of the growth factor content. To overcome this, Reginensi and colleagues evaluated for the first time decellularized bio-inks from different sections of the brain (i.e., cortex, cerebellum, and remaining areas) using both mechanical and chemical decellularization protocols [133]. Intriguingly, they found that the chemical method (mixture of different enzymes) promotes greater differentiation, probably because it allowed the conservation of the biochemical components of the cerebral ECM. Moreover, the authors show differences in neuronal maturation depending on the region of the brain used to produce the scaffolds [133]. One of the main advantages of decellularized scaffolds is the possibility to chemically modify them to obtain better features. To this end, Beachley and colleagues developed a decellularized brain tissue scaffold crosslinked with glycosaminoglycans, to facilitate ECM hydrogel formation without a disruptive enzymatic digestion process [134]. Moreover, they proved that using the ECM from different tissues at various concentrations allowed the gelation kinetics and mechanical properties to be easily tuned to offer the possibility of numerous in vivo and in vitro applications with different property requirements [134]. Decellularized scaffolds can also help the differentiation of glial cells. Cho and colleagues utilized decellularized human brain tissue and found an enhancement of the differentiation of induced Pluripotent Stem Cells (iPSCs) myelin-expressing oligodendrocytes, which improved the electrophysiological properties of induced neural cells [135]. Decellularized scaffolds could also offer new opportunities for therapeutic applications in regenerative medicine. For example, Lin and colleagues used brain-derived decellularized scaffold added with basic fibroblast growth factor (bFGF), which is studied as a potential agent for PD. They found that the presence of bFGF not only enhanced the viability of PD model cells but also improved the behavioral recovery and positive expressions of neurotrophic proteins in PD rats [136]. The therapeutic potential of decellularized bio-inks was also evaluated by Tukmachev and colleagues, who assessed the effects of both porcine spinal cord and porcine urinary bladder decellularized injectable hydrogels in an in vivo model of acute SCI. They found that both types of hydrogels integrated into the lesion, stimulating neovascularization and axonal ingrowth into the lesion. On the other hand, they found a rapid degradation of the hydrogel [137]. In conclusion, decellularized scaffolds represent an interesting innovative technique for the generation of a new neurodegenerative model and for the development of new therapeutic approaches, but further studies must be conducted in order to resolve the actual issues. 

### 4.2. Hydrogel-Based Biomaterials

One of the most relevant groups of biomaterials that can be chosen are hydrogels because of their high biocompatibility, their chemical features, and their similarity to the ECM [138]. One of the first attempts to generate a layered brain-like structure is represented by the work of Lozano and colleagues [139]. The group combined mouse primary cortical neurons with a bio-ink composed of gellan gum functionalized with the arginine-glycine-aspartic acid (RDG) peptide. They demonstrated that the bio-ink strongly supported the neural proliferation and cell–cell communication [139]. The ability to form discrete multiple layers opened the opportunity to reproduce accurate 3-D human brain models [139]. Indeed, another important parameter to evaluate the best approach in modeling neurodegenerative diseases is the specific cell type. For preliminary studies, such as the biocompatibility of the hydrogel, immortalized cell lines are used, such as rat pheochromocytoma cell lines (PC12) [140] and the human neuroblastoma cell line (SH-SY5Y) [141]. Such models can help investigate the effects on the maturation/differentiation of composite hydrogels or other biomaterials. For example, some studies were conducted on conductive hydrogels [142,143,144], nanofibrous scaffolds [145,146,147], and self-assembling peptide scaffolds [148,149]. Although such cell lines are important for preliminary investigations, to obtain a realistic in vitro model, it is essential to use stem cell-derived mature neurons. In the last years, the possibility to reprogram somatic cells into iPSCs allowed patient-derived neural cells to be obtained, opening new possibilities in the field of tissue engineering. The use of iPSCs with hydrogels can offer some advantages. For example, Zhang and colleagues found that a hyaluronic acid-based hydrogel accelerates the maturation of iPSCs into neural progenitor cells, because of the similarity with the brain tissue [150]. Although iPSCs were 3-D bioprinted and then differentiated into the hydrogel, some evidence suggests that is better to directly bioprint iPSC-derived neural stem cells (NSCs) [151]. This specific fabrication technique was used, and many phenotypic aspects were investigated, such as the analysis of neurite extension and neural maturation on polyethylene glycol [152] or 3-D gelatin methacrylate [153] hydrogels. Moreover, iPSC-derived mature neurons were bioprinted and cultivated on hydrogel scaffolds, and many read-outs were evaluated, such as the expression of integrins, the formation of a complex network, and the expression of synaptophysin along the neurites [125]. iPSCs can also be differentiated into glial cells, which proved to play a pivotal role in the pathogenesis of neurological disorders. For example, in 2020, Nazari and colleagues differentiated iPSCs into oligodendrocytes in a fibrin-based hydrogel, demonstrating a better proliferation in the 3-D culture system with respect to monolayer culture [154]. In particular, the results reported demonstrated that fibrin hydrogels provide a metabolically active microenvironment for cells, mimicking specific features of native tissue.

All these studies provide a strong evidence that hydrogel-based scaffolds can mimic in a very realistic way the neural ECM, opening new perspectives for the study of neurological disorders and for the development of new therapeutic approaches.

## 5. The Role of Scaffolds in Developing Regenerative Therapies for Neurodegenerative Diseases

Amongst all the organs, the human brain possibly represents the biggest challenge in terms of the modelling and development of therapeutic strategies. Its structural complexity and the inability to retrieve samples without highly invasive, and often unfeasible, approaches represent the major limitation in the study of physiological and pathological brain activity. The brain’s inability to regenerate also implies that damages are often irreversible, and, so far, no cure for neurodegeneration has been found. For this reason, there is an unmet need for new strategies aiming at modeling and curing neurodegenerative diseases. The use of scaffolds becomes of great relevance in this field, as they can be used to mimic the brain’s morphology and function, to improve cellular growth for transplantation means, to improve drug delivery, and even to be directly transplanted at the lesion site. 

**Figure 3:**
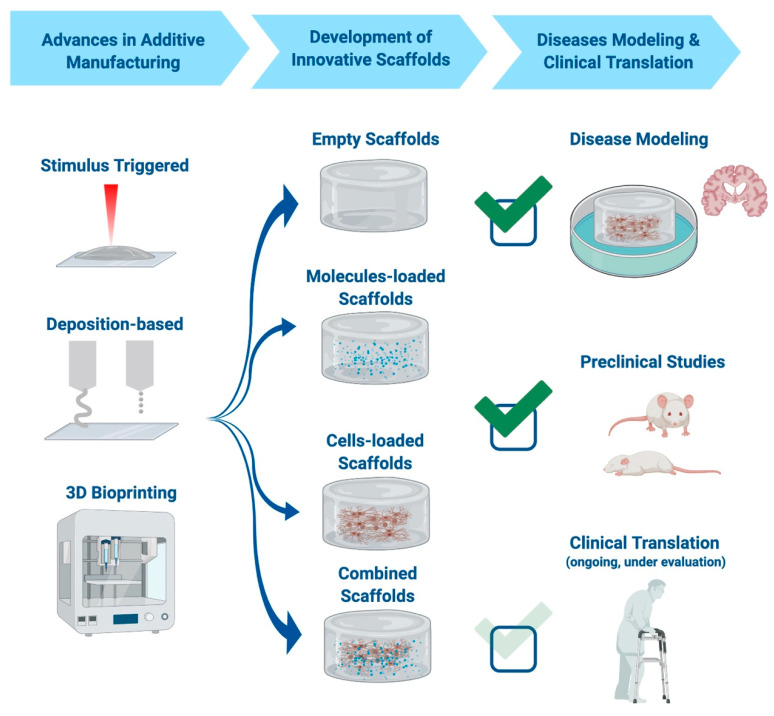
Use of additive manufacturing techniques in neurodegenerative diseases. Scaffolds can be either printed or bio printed, and embedded with molecules, cells, or even a combination of the two to increase their therapeutic efficiency. Recent advances in production technologies have shown a relevance for these techniques in disease modeling and preclinical models of neurodegenerative diseases. Current efforts are focusing on the development of safe and efficient strategies for human clinical translation. Made in ©BioRender—biorender.com.

As mentioned above, the main unmet clinical need in neurodegenerative diseases is the lack of successful replacement therapy for damaged brain tissue. Although it is worth looking at recent advances made in specific diseases, some common patterns can be highlighted. Indeed, in all cases, it is possible to discriminate between the transplantation of empty scaffolds, of scaffolds carrying specific therapeutic agents, of cell-loaded scaffolds, or scaffolds combining cells and molecules. The use of scaffolds allowed significant advances in disease modeling, as explained in the previous paragraph, and when focusing on diseases therapy, most research is currently focused on pre-clinical studies. Indeed, although there is a great potentiality for scaffolds’ use, it is of course firstly necessary to evaluate the safety and efficacy of each scaffold in pre-clinical models [155,156,157,158,159,160,161,162,163,164,165,166]. Indeed, a lack of toxicity and an improvement in diseases’ hallmarks are necessary aims that need to be fulfilled before moving on to clinical practice. Indeed, the implant of scaffolds in humans is currently limited, with one of the few examples being represented by in the Neuro-Spinal Scaffold (InVivo Therapeutics Corp.) for the treatment of SCI [167,168]. All these aspects are reported in Figure 3.

Similarly to disease modeling, the most promising and commonly used material in the treatment of these diseases is hydrogel for its increased bioavailability and characteristics, which mimic those of the ECM well. The most commonly used approach is the delivery in situ of pharmaceutical agents already known for their therapeutic potential in the specific disease. The use of scaffolds in this case improves the therapeutic efficacy as the contained agents are “protected” by the hydrogel and thus present a decreased degradation rate [156,169]. In a similar manner, cell-loaded scaffolds can bridge stem cell therapy with tissue engineering, improving the delivery of stem cells to the lesioned site [159,160,161,162]. Even if most of the first developed agents contained one therapeutic agent, combinatory approaches now aim to deliver both drugs and cells, or even different drugs contained in the same scaffold but released at different times after scaffold delivery [170]. This is a great advancement as often just one therapeutic agent is not enough to re-create the physiological situation that was present before the lesion began. In some cases, it is also possible to have a controlled release of the therapeutic agent, allowing its constant and prolonged delivery. Even so, more recent works are investigating the potentiality of different materials and structures, such as it is the case for electrospun polymers in PD [171,172]. 

### 5.1. Alzheimer’s Disease

Alzheimer’s disease (AD) is the main cause of dementia worldwide, characterized by a decline in cognitive functions and subsequent memory loss [173,174]. The two cellular hallmarks of the disease are the presence of extracellular amyloid plaques and intracellular neurofibrillary tangles (characterized by tau hyperphosphorylation) [175]. Even so, very little is known about the causes that lead to AD onset, and to this day, there is no curative therapy for the disease [174,175]. There is thus a need to evolve from current pharmacological strategies, which, for now, are only a symptomatic remedy [174,176]. A therapeutic advancement that is gaining more and more relevance this day is the use of scaffolds, advantageous for both drug delivery and for promoting stem cell delivery and survival in the hostile AD microenvironment [176]. In this context, hydrogels are of key importance for the delivery of therapeutic agents, in order to increase their bioavailability [156]. Examples include biodegradable microspheres loaded with huperzine A (a natural acetylcholinesterase inhibitor) [177], microemulsion loaded with tacrine [178,179], microspheres optimized to deliver Nerve Growth Factor (NGF) [180], poly(lactic-co-glycolic acid) (PLGA) nanoparticles loaded with estradiol or tempol [181,182], and hiolated chitosan hydrogels loaded with donepezil [183]. Innovative developments combine more therapeutic aspects, such as a novel peptide-based hydrogel, which contains a peptide that stabilizes the microtubules, associated to a neuroprotective action, and able to promote neurite outgrowth of neuron cells [184]. This hydrogel is also able to encapsulate curcumin and release it slowly, and although this hydrogel has not been tested yet in vivo, it could represent a promising therapeutic strategy [184]. Recently, a combinatory approach has also been tested, combining liposomes with hydrogels and delivering an active pharmaceutical ingredient, in order to improve bioavailability [185].

Furthermore, scaffolds can be combined with stem cell therapies to improve functional outcomes in AD [176]. An example of this is the scaffold made of RADARADARADARADA (RADA16) peptide combined with part of the laminin sequence, which when transplanted with NSCs in a rat model of AD protected cells against apoptosis and promoted neuronal differentiation, resulting in an improvement of behavioral outcomes [155].

### 5.2. Parkinson’s Disease

Parkinson’s disease (PD) is the second most common neurodegenerative disorder, characterized by the loss of dopaminergic neurons in the substantia nigra pars compacta (SNpc) [186] and the presence of alpha synuclein, which aggregates in toxic components termed Lewy bodies [187]. The first line of therapy for the treatment of PD is dopaminergic agonists (such as L-3,4-dihydroxyphenylalanine, L-DOPA, combined with other tested drugs), which only provide a symptomatic remedy and allow treatment for at least 5 years [188]. Other pharmacological agents and even stem cell therapy are being considered for the treatment of PD, but a disease-modifying therapeutic agent has not yet been developed [189]. A number of works have investigated the potentiality of hydrogels in the delivery of therapeutic agents (both pharmacological and biologicals) in in vivo models of PD [156,157,158]. Indeed, the delivery of pharmacological agents, which have been proved effective in the attenuation of PD’s symptoms, can be potentiated with hydrogels [156,169]. These scaffolds “protect” the delivered neurotrophic agents (e.g., Glial cell-derived neurotrophic factor GDNF, NGF) and promote a controlled and localized release, as it is possible to determine their decay. Examples include the development of systems aimed at improving the delivery of the therapeutic agent: A hydrogel-based system aimed at improving transdermal dopamine delivery [157] and a biodegradable polymer matrix releasing dopamine in the striatum of a hemi-parkinsonian animal model [190]. Systems have been developed for the administration of neurotrophic factors, already partially studied as treatment of the diseases, such as GDNF-loaded microspheres stereotaxically implanted in brain of PD-affected animals [158,191,192]. Hydrogels have also been used for the delivery of less canonical therapeutic agents, such as Tat-fused protein Heat Shock Protein 70 (Hsp70) [193], activin-B [194], or even the secretome of mesenchymal stem cells [195]. In comparison to canonical drug delivery, cell therapy is also a promising approach for PD therapy [196,197]. Indeed, another bioengineering application is the use of an innovative strategy to improve the efficiency of hydrogel in cellular transplantation and delivery through the addition of neurotrophic factors (such as BDNF), adhesion molecules, or a combined system of hydrogels and nanoparticles [198,199,200,201,202,203,204]. Hydrogels can also be used to ameliorate the differentiation of iPSCs to a dopaminergic phenotype, which could prove useful in drug screening and disease modelling [205].

Aside from hydrogels, very few studies investigated the potentiality of scaffolds in the therapy of neurodegenerative diseases. Carlson and colleagues developed three-dimensional microtopographic scaffolds using tunable electrospun microfibrous polymeric substrates that appear to promote in situ stem cell neuronal reprogramming, neural network establishment, and support neuronal engraftment into the brain. The authors aimed to develop a mini-neurocircuitry composed of excitatory dopaminergic neurons, which could have a profound impact in the amelioration of PD symptoms [171]. Another novel 3-D nanofiber scaffold has been developed using electrospun PAN, a pure carbon-based polymer, and Jeffamine^®^ polymer-infused PAN. Both scaffolds are capable of promoting survival and proliferation of SH-SY5Y and U-87MG cells, and when these were incubated with PD-mimicking agents, cell survival inside the scaffold was increased with respect to 2-D culture conditions [172]. 

### 5.3. Amyotrophic Lateral Sclerosis

Amyotrophic lateral sclerosis (ALS) is the most prevalent motor neuron disease, characterized by the progressive loss of upper and lower motor neurons [206]. Life expectancy is 2-5 years after the first diagnosis, with death being caused in the majority of cases by muscle atrophy and paralysis, which becomes life threatening when respiratory muscles are involved [207]. Other than the canonical neuronal dysfunctions observed in neurodegenerative diseases (e.g., oxidative stress, protein aggregation, loss of synaptic activity), a strong contributor for the development of ALS pathogenesis is represented by astrocytes, which in the disease lack the ability to support neurons [208,209,210]. For this reason, and for the complex multi-cellular system present in ALS, 2-D cultures are not sufficient to recapitulate the disease course. Even so, a very limited number of 3-D scaffold-based therapies have been developed, and future studies should focus on their development in order to gain more in-depth insight in disease pathogenesis [210,211]. 

Gingras and colleagues developed a 3-D tissue engineered model to study motor neuronal axonal migration and myelination. In this model, mouse spinal cord motor neurons were seeded on a collagen sponge populated with Schwann cells and fibroblasts. The model permitted study of the fundamental characteristics of motor neurons, such as neurite outgrowth and spontaneous myelination. Even if performed in healthy cells, the model could prove to be relatable and applicable for the study of the pathogenesis of motor neuron diseases [212]. The possibility of combining healthy and diseased cells (such as the ones that can be obtained from ALS patients) allows the identification of the effects of neuronal or astrocyte toxicity in ALS and to create more representative disease models [211]. Another aspect of ALS pathology that needs to be investigated is the muscles’ role in the disease. A very interesting 3-D model was represented by primary muscle cultures obtained from human control subjects and ALS patients, embedded in a collagen gel. The model also allowed the study of gel contraction and the aim was to study in vitro the effect of muscle stretching on mRNA expression in diseased muscles cells [213,214]. The gold standard model that recapitulates all of the disease aspects observed in ALS would have to be a whole spinal cord organoid obtained from ALS patient-derived iPSCs [215]. Although this has not yet been developed, recent advances allowed the development of 3-D-engineered spinal cord models [216,217]. In particular, Bowser and Moore developed a combined microphysiological system, where spinal cord spheroids are fabricated using magnetic nanoparticles and then positioned in a 3-D hydrogel construct using magnetic bioprinting [217]. So far, no studies are present that have investigated scaffolds for the treatment of ALS. Even so, devices, such as the “syringe-injectable nano-scale electronic scaffolds”, which can be used to monitor neural activity, stimulate tissues, and promote neuronal regeneration, could be of great relevance in the treatment of the disease [218]. Lastly, one work developed a technique for intrathecal transplantation of glial progenitor cell-loaded hydrogels through Magnetic Resonance Imaging (MRI)-guided delivery in a naturally occurring ALS-like disease in dogs. The procedure was found to be safe and the embedded cells were successfully placed [219]. 

### 5.4. Acute Ischemic Stroke

Acute ischemic stroke (AIS) is caused by a transient or permanent reduction in cerebral blood flow, generally caused by the occlusion of a cerebral artery, an embolus, or local thrombosis [220]. The consequences of AIS are hypoxia, an increase in radical oxygen species, and an excessive inflammatory response, which can lead to long-term consequences [220,221]. AIS leads to a loss of brain tissue, which is not regenerated, even if neurogenesis partially occurs, and this is due to the lack of structural support and the fact that the tissue undergoing injury creates a boundary to avoid damage expansion into the healthy tissue [222]. It is for this reason that bio-scaffolds could prove useful for the therapy of this disease, providing structural support [222,223]. The first evidence that supported the potentiality of scaffold use in AIS was determined after the transplantation of NSCs on polymeric scaffolds, with promising results [159,160,161,162]. Hydrogels improved the interaction between NSCs and the host tissue, through neuronal differentiation, re-formation of cortical tissue, increased connectivity, reduced inflammation, and reduced scarring [159]. Similar results were obtained when NSCs were transplanted with polymerized allylamine (ppAAm)-treated PLGA scaffolds [224] and xenogenic extracellular matrix bio-scaffolds [225]. Other types of cells, such as iPSCs [152,226] and bone marrow mesenchymal stem cells, can be transplanted together with other kinds of scaffolds (e.g., gel-like scaffold from plasma) [227]. Limitations with these approaches included the non-homogeneity of the scaffold and the need for vascularization [222,224,225]. Another scaffolding approach used for the treatment of AIS is 3-D bioprinting [228]. In the case of AIS, biomaterials can favor cellular integration and reduce the immune response [228,229,230]. 

Additionally, in this case, hydrogels can be used for the delivery of pharmacological agents, which proved beneficial for stroke, such as erythropoietin (EPO), a cytokine found to promote neurogenesis after AIS [223,231,232]; or vascular endothelial growth factor (VEGF), capable of inducing structural protection after AIS [233,234]; and brain-derived neurotrophic factor (BDNF) [235]. Hydrogels have also been developed for sustained delivery of cyclosporine A, stimulating neurogenesis in the damaged tissue of rodent brains [236,237]. Combinatory approaches can also be used, such as the co-administration of EPO and epidermal growth factor (EGF) [238,239] or of VEGF and angiopoietin-1 [240]. In the case of AIS, hydrogels have also been combined with proteins, proving efficient in the treatment of the disease [223]. Specifically, they have been linked to genipin [241] and fibrin [242,243,244,245]. There is a need to develop a strategy to correctly implant hydrogels, and also in this case, as reported for ALS, an MRI-guided approach could be adopted [225,246]. It is very interesting to report that in the case of AIS, bio-scaffolds could also serve as a preventive tool, especially in cases where atherosclerotic plaques are already present and represent a strong risk factor for AIS insurgence [228]. Although no trials have yet been done, the combination of computational fluid dynamics based on patients’ imaging and scaffolds with a patient-specific 3-D geometry could allow first the testing and then the implantation of devices able to correct the arterial flow [228]. 

### 5.5. Spinal Cord Injury

SCI is a devastating disease caused by high-energy trauma, which leads to severe neurological dysfunctions [247,248]. Patients suffer from partial or complete limb paralysis, associated with sensory dysfunction, urinary incontinence, or gastrointestinal dysfunctions [247,248]. To this day, there is no curative therapy, and treatments are aimed at reducing secondary degeneration with high-dose corticosteroids, surgical stabilization, and decompression [249,250]. It is especially during surgical stabilization that scaffolds could prove to be efficient, maybe improving current lines of therapy. The concept of a solid matrix providing support in SCI has already been tested [251,252] and it is worth focusing on both the potentiality of scaffolds alone and that of scaffolds combined with therapeutic agents (stem cells, drugs, growth factors, etc.). In the initial approach, synthetic scaffolds (e.g., physical chitosan microhydrogels) were used to bridge the extremities of damaged spinal cords to promote regeneration and connectivity [253,254,255]. More recently, they were implemented with drugs, growth factors, and even stem cells in order to obtain a controlled release of drugs, stimulation of endogenous regeneration, and local secretion of neurotrophic factors and stem cell delivery [163,164,165,166]. Scaffolds that can be used for SCI were illustrated in depth by Zhang and colleagues and include: Biodegradable synthetic polymer scaffolds (PCL, PLA, PLGA, PEG), non-biodegradable synthetic polymer scaffolds (PHEMA, PHPMA, PAN/PVC, conductive polymers), and natural polymer scaffolds (collagen, chitosan, alginate, fibrin) [256]. An interesting and promising technology is represented by graphene oxide (GO) 3-D nano-structured scaffolds, able to stimulate neuronal differentiation thanks to unique electro-physico-chemical properties [257,258,259,260]. To this end, a key player in providing structural support could also be the physiological tissue, such as adipose tissue, which has proved promising in the treatment of the disease [261,262]. Studies showed that the beneficial effects were given not only by the structural support but also by the fact that mesenchymal stem cells present inside the adipose tissue promoted regeneration and recovery of function, and reduced the inflammatory response [261,262].

Amongst the neuroprotective factors incorporated in hydrogels, promising results have been obtained with the delivery of neurotrophin-3 [263,264,265], nerve growth factor [266], BDNF [267], and neuregulin [268]. One of the most innovative research works in this field required a complex combinatorial system of growth factors and bio-scaffolds. It was based on a two-step protocol, with different factors being modulated before and after the lesion, in order to allow for axonal re-growth. The protocol began with a time-dependent administration of adeno-associated viral vectors to reactivate neuroregeneration (through downregulation of phosphatase and tensin homologue; upregulation of osteopontin, insulin-like growth factor 1, and ciliary-derived neurotrophic factor). After SCI was induced, the protocol required the injection of biomaterials (made of diblock copolypeptide hydrogels) delivering fibroblast growth factor 2, EGF, glial cell-derived neurotrophic factor (GDNF), and integrin-blocking antibody [170]. Other than compounds, bio-scaffolds can also in this case be used to deliver stem cells, most frequently human mesenchymal stem cells, widely used in the treatment of the disease [269,270,271], but scaffolds complexed with NPCs have also been developed [166,272,273]. It is worth mentioning that in the case of SCI, biomaterials (in particular the Neuro-Spinal Scaffold - InVivo Therapeutics Corp.) have also been used in clinical practice [167,168]. The trial has completed recruitment and is currently in the follow-up phase (NCT02138110), but the case of a patient who underwent surgery has been published, with improvements in neurological function and no procedural complications [168].

The results of the recent advances reported above are summarized in Table 2. These include scaffolds fabricated with additive manufacturing techniques along with innovative discoveries aimed at improving hydrogel scaffolds, especially relevant in neurodegenerative diseases’ therapy.

## 6. Conclusions

The characteristics of pathogenesis and lack of regenerative therapeutic approaches in neurodegenerative diseases implicates the need to develop new innovative therapeutic strategies. Additive manufacturing techniques have gained more and more relevance, proving the great potential of the fabrication of precision scaffolds, which could enhance therapeutic efficiency and even provide patient-specific 3-D scaffolds. This, together with the specific fabrication of scaffolds with precise geometry and structure, could prove of great importance in the field of regenerative medicine and neurodegenerative disease therapy.

## Figures and Tables

**Table 1:** Summary of additive manufacturing techniques for scaffold fabrication.

Fabrication Approach	Fabrication Technique	Principle of Operation	Resolution	Advantages	Limitations
Stimulus-Triggered	3-D Printing (particle bonding)	Binder solution ejection on powder bed	~300 µm	Mix of powder Controlled architecture	Low spatial resolution Post-fabrication treatment Pore size
	SLS/SLM	Locally powder bed sintering/melting	~50 µm	No supporting structure No organic solvent Materials availability	High Temperature Poor surface accuracy Poor interconnection control
	SLA	Photopolymerization of UV-curable resin at surface	1 µm	Low cost equipment High processing speed	Polymerization effects Post-curing treatment
	2PP	Photopolymerization of UV-curable resin at laser focus	100 nm	Higher resolution No controlled environment	Polymerization effects
Deposition-based	FDM	Fused material extrusion/solidification upon cooling	~250 µm	No toxic solvents Materials availability	Low spatial resolution High temperatures Low dimensional accuracy
	MJP	Droplets deposition of UV-curable resin	50–1 µm	High spatial resolution	Expensive materials Rheology control
3D Bioprinting	InkJet Bioprinting	Bio-Ink droplets deposition	300–50 µm	Single cell encapsulation	Low spatial resolution Low viscosity upper limit
	DIW	Bio-Ink extrusion	~200 µm	High processing speed High cellular densities Larger structures fabrication	Low spatial resolution Apoptotic effect (for mechanical-based system)
	LAB	Laser induced Bio-Ink droplets deposition	<20 μm	Good spatial resolution High bioactivity	Rheology control

**Table 2:** Summary of therapeutic agents delivered with scaffolds to treat neurodegenerative diseases.

Disease	Molecules Delivery	Cells Delivery	Combined Delivery
Alzheimer Disease	Huperzine A, Tacrine, Nerve Growth Factor, Estradiol, Tempol, Donezepil [177,178,179,180,181,182,183]	Neural Stem Cells [155]	Curcumin + Neuroprotective peptide, Liposomes + hydrogels [184,185]
Parkinson’s Disease	Dopamine, Glial Cell-Derived Neurotrophic Factor Hsp70, Activin-B, Mesenchymal Stem Cells’ secretome [190,191,192,193,195]	fetal Neural Stem Cells, human Embryonic Stem Cells, Mesenchymal Stem Cells, induced Pluripotent Stem Cells [200,201,202,203,205]	Dopaminergic neurons + Glial Cell-Derived Neurotrophic Factor, Neural Cells + Brain-derived neurotrophic factor, Hydrogels + Nanoparticles [157,199,204]
Amyotrophic Lateral Sclerosis	N/A	Glial Progenitor cells [219]	N/A
Acute Ischemic Stroke	Erythropoietin, Vascular endothelial growth factor, Brain-derived neurotrophic factor, Cyclosporine A, Genipin, Fibrin [231,233,234,235,236,237,241,242,243,244,245]	Neural Stem Cells, Neural Precursor Stem Cells, induced Pluripotent Stem Cells, Bone Marrow Mesenchymal Cells [159,160,161,162,224,225,226,227,229]	Erythropoietin + Epidermal Growth Factor, Vascular endothelial growth factor + Angiopoietin [238,239,240]
Spinal Cord Injury	Neurotrophin-3, Nerve Growth Factor, Brain-derived neurotrophic factor, Neuregulin [263,264,265,266,267,268]	Human Mesenchymal Stem Cells, Neural Precursor Stem Cells [166,269,270,271,272,273]	Viral vectors + basic fibroblast growth factor+ Epidermal Growth Factor + Glial Cell-Derived Neurotrophic Factor + integrin-blocking antibody [170]
